# Body size, nutritional state and endocrine state are associated with calving probability in a long‐lived marine species

**DOI:** 10.1111/1365-2656.70068

**Published:** 2025-06-12

**Authors:** Enrico Pirotta, Leslie New, Alejandro Fernandez Ajó, K. C. Bierlich, Clara N. Bird, C. Loren Buck, Lisa Hildebrand, Kathleen E. Hunt, John Calambokidis, Leigh G. Torres

**Affiliations:** ^1^ Centre for Research into Ecological and Environmental Modelling University of St Andrews St Andrews UK; ^2^ Department of Mathematics, Computer Science and Statistics Ursinus College Collegeville Pennsylvania USA; ^3^ Geospatial Ecology of Marine Megafauna Lab, Marine Mammal Institute, Department of Fisheries, Wildlife and Conservation Sciences Oregon State University Newport Oregon USA; ^4^ Department of Biological Sciences Northern Arizona University Flagstaff Arizona USA; ^5^ Smithsonian‐Mason School of Conservation & Department of Biology George Mason University Front Royal Virginia USA; ^6^ Cascadia Research Collective Olympia Washington USA

**Keywords:** Bayesian state–space modelling, calving probability, early warning signals, gray whales, health, Pacific Coast Feeding Group, population consequences of disturbance

## Abstract

1. Life‐history performance of individuals in wildlife populations emerges from the interplay between the multiple processes that constitute an animal's health. Monitoring and modelling indicators of health can thus provide a way to assess and forecast the status of a population before its abundance changes.
2. In this study, we develop a Bayesian state–space model that links multiple health indicators (representing energy, endocrine and morphometric status) and resulting female calving probability, using an 8‐year dataset of repeated sightings, morphological measurements, faecal sampling and offspring observations of gray whales (*Eschrichtius robustus*) belonging to the Pacific Coast Feeding Group.
3. Model results indicate that calving probability emerges from the combined effect of a female's structural body size and available energy reserves, while also showing a weak negative correlation with glucocorticoid levels prior to pregnancy. Assessment of population age structure suggests that the number of individuals in younger age classes is smaller than expected for a growing or a stable population, which, together with decreasing body size, could indicate an impending decline in this group. Model development was made possible by the collection of high‐resolution, longitudinal data on individuals, although several mechanistic assumptions were imposed by the relatively short time series (8 years), influencing the results.
4. Our modelling approach could inform similar efforts in other long‐lived species where population dynamics cannot be easily monitored. Ultimately, models of wildlife health and vital rates can support assessments of the population‐level consequences of multiple stressors, a key goal for management and conservation across systems and jurisdictions.

## Graphical abstract

The study develops an approach to model multiple health indicators in long‐lived species. Gray whale calving probability emerges from the combined effect of structural body size and available energy reserves. Activation of the hypothalamic–pituitary–adrenal axis may also affect reproduction. This group of gray whales shows early warnings of impending population decline.

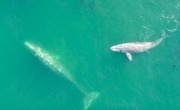

## 1 INTRODUCTION

Long‐lived, wide‐ranging species present many challenges to management and conservation efforts. Monitoring their population size requires large, long‐term investments and the resulting data often do not grant sufficient statistical power to detect changes in abundance on a management‐relevant timescale (Coulson et al., 2001; Taylor et al., 2007; White, 2019). This long‐standing problem has stimulated the development of tools to assess and forecast population status before it manifests into a decline, including mechanistic modelling approaches to predict a population's trajectory (e.g. Deangelis & Mooij, 2005), as well as the identification of data streams that can be feasibly monitored as warning signals (Clements & Ozgul, 2018; Scheffer et al., 2009). For example, Cerini et al. (2023) described a succession of changes under adverse conditions, from altered behaviour to morphological changes, reduction in reproductive rates and, finally, abundance decline. A similar paradigm was proposed by Eberhardt (2002), suggesting that, as resources become limiting, juvenile survival drops, followed by slower growth and delayed age of first reproduction, reduced reproductive rates and, lastly, lower adult survival. Necessarily, developing such modelling and monitoring tools therefore requires shifting the focus away from the population as a whole and onto individuals.

The life‐history performance of individuals emerges from the complex combination of multiple underlying processes. These processes integrate in the concept of individual health, which has been defined as “the ability of an organism to adapt to and manage threats to survival and reproduction” (Tyack et al., 2022). In practice, health can be represented as a series of indicators that track the status of different physiological systems (Tyack et al., 2022). Modern technology is improving our ability to measure health indicators consistently and across a large proportion of a population (e.g. via blood or morphometric analyses; Kophamel et al., 2022) and, crucially, to link them to life‐history outcomes (e.g. Schwacke et al., 2024). However, for most long‐lived species, some degree of mechanistic information is required to improve the precision and predictive power enabled by available data (Pirotta et al., 2022).

Research on marine mammals, a polyphyletic group with diverse behaviour, physiology and ecology, has long acknowledged the challenges of collecting suitable data to inform conservation. Most species tend to be long‐lived, wide‐ranging and hard to access, which makes it difficult to monitor their status, quantify the effects of stressors and, ultimately, manage them effectively (Taylor et al., 2007). Nonetheless, several international jurisdictions mandate their protection from the many stressors they are exposed to (e.g. fishing gear bycatch, vessel collisions, pollutants, wildlife tourism, noise and industrial development) in a rapidly changing climate that is affecting their habitats (Nelms et al., 2021). The Population Consequences of Multiple Stressors conceptual framework links individual behavioural and physiological responses to effects on vital rates and population dynamics via changes in health (National Academies, 2017; Pirotta et al., 2022; Tyack et al., 2022). Most work to date has focused on the behaviour–bioenergetic axis, which characterises an individual's health in terms of its energy reserves, allowing the conversion of behavioural patterns under disturbed vs. undisturbed conditions into a common currency (Keen et al., 2021; Pirotta et al., 2018). Other axes (e.g. effects mediated by the endocrine or immune systems) are also important, but the associated data collection and model development are more challenging (National Academies, 2017).

Previous work on North Atlantic right whales (*Eubalaena glacialis*) has shown how the Population Consequences of Multiple Stressors concepts can be operationalised for baleen whales using state–space modelling approaches (Pirotta, Schick, et al., 2023; Schick et al., 2013). State–space models can accommodate both knowledge of the underlying mechanisms and emergent data streams (Auger‐Méthé et al., 2021). Across taxa, these methods have been extensively used in ecology to model animal population dynamics (Buckland et al., 2004), spatial distribution (Royle & Kéry, 2007) and movements (Patterson et al., 2008). However, their application to model the time series of individual traits has been more limited (e.g. for individual growth; Brooks et al., 2017), with even rarer examples where the underlying state variable (usually body mass) was linked to vital rates (e.g. New et al., 2014; Pigeon et al., 2022). In right whales, unobserved individual health is estimated over time, integrating the effects of multiple stressors and driving vital rates (Pirotta, Schick, et al., 2023; Schick et al., 2013). However, this species has been studied for multiple decades, resulting in a uniquely large dataset to inform modelling, and is Critically Endangered, implying that life‐history heterogeneity among individuals is more easily quantifiable; most species do not meet these criteria but still face threats that can affect their vital rates.

Here, we focus on a subgroup of ~212 gray whales (*Eschrichtius robustus*) belonging to the Pacific Coast Feeding Group (PCFG) to test the applicability of these methods to other species. This subgroup has been studied since the mid‐1990s (Calambokidis et al., 2002; Harris et al., 2022), with detailed data collected since 2016 on diet, behaviour, morphology and physiology along the central Oregon coast, USA (e.g. Bird et al., 2024; Hildebrand et al., 2021; Lemos, Burnett, et al., 2020; Pirotta, Bierlich, et al., 2024; Pirotta, Fernandez Ajó, et al., 2023). PCFG whales migrate to coastal waters between northern California (USA) and Vancouver Island (Canada) as foraging grounds, located at lower latitudes compared to the broader Eastern North Pacific population. Conception is thought to occur during the southbound migration in December–January, and gestation lasts approximately 13 months (Rice & Wolman, 1971). Most births occur in the Mexican breeding grounds during January, followed by ~7 months of lactation that usually ends in July–August (Rice & Wolman, 1971). While the Eastern North Pacific population is believed to be at carrying capacity and has been undergoing boom‐bust cycles driven by environmental variation (Stewart et al., 2023), PCFG abundance is thought to be stable (Barlow et al., 2024; Harris et al., 2022). However, PCFG whales are smaller and in poorer body condition than Eastern North Pacific gray whales (Bierlich et al., 2023; Torres et al., 2022), and their body size has been decreasing since the early 2000s (Pirotta, Bierlich, et al., 2024)—a pattern that has been associated with declining calving probability in other whale species (Pirotta, Tyack, et al., 2024) and thus could be an early warning signal of potential population decline (Clements & Ozgul, 2016).

In this study, our goal is to develop a state–space model that captures multiple aspects of PCFG gray whale health to assess their influence on an individual's vital rates. We hypothesised that the shorter temporal span (8 years vs. 5 decades) and higher resolution of available data compared to North Atlantic right whales would impose different modelling decisions. In particular, given the limited data on deaths in the PCFG during our study period, we focus on female calving probability, which is likely a more relevant and tractable vital rate in a non‐declining, long‐lived population. Overall, this work demonstrates the development of Population Consequences of Multiple Stressors methods in contexts where data collection spans less than one decade and is limited to a subset of the population. The resulting approach could inform similar modelling exercises in other systems involving species where population dynamics cannot easily be monitored.

## 2 MATERIALS AND METHODS

### 2.1 Data collection and processing

Visual surveys targeting PCFG gray whales were conducted along central Oregon, USA (departing from Newport, OR, 44.6368° N 124.0535° W) from a 5.4‐m research vessel (Pirotta, Fernandez Ajó, et al., 2023), between late May and mid‐October over 8 years (2016–2023). Individual whales were photo‐identified from field photographs using catalogues for the PCFG held by the Marine Mammal Institute at Oregon State University and Cascadia Research Collective (Olympia, WA, USA). Sex was derived from observations with dependent calves or genetic analyses (Lang et al., 2014; Lemos, Olsen, et al., 2020). We recorded the occurrence of calves and the identity of their mother (assumed to be the individual they were swimming near), and we obtained additional information on calf occurrence in our study years from other research groups in the PCFG Consortium (https://pcfgconsortium.org/). Only one PCFG individual was observed dead in the study period; therefore, we decided to focus our analysis on reproduction, ignoring processes associated with survival.

Aerial images of individual whales were collected using drones as described in Pirotta, Bierlich, et al. (2024). The model for individual growth in Pirotta, Bierlich, et al. (2024) was used to estimate individuals' total length in each year, with uncertainty, from aerial imagery. Their model also returns estimates of individual age in years, either corresponding to a known age when whales were first sighted as calves by contributors to the Consortium database, or estimated from a minimum estimate based on time elapsed from the first sighting. This estimation is informed by the growth model and each individual's photogrammetric measurements (see Pirotta, Bierlich, et al., 2024 for details and validation). The model was also extended to concurrently compute body area index (BAI; Supporting Information), a unitless and length‐standardised metric of body condition (Burnett et al., 2019). Only individuals with at least one BAI measurement during the study period were retained in subsequent analyses. We investigated the population's apparent age structure by plotting the number of individuals of a given age (known or estimated) in 2023, as a coarse indication of population status to compare against the calving probabilities estimated from the model.

Faecal samples from individual whales were collected as described in Pirotta, Fernandez Ajó, et al. (2023), which also report the details of sample storage and preparation and hormone extraction. Commercial enzyme immunoassay kits for cortisol (#ADI‐900‐217,071), progesterone (#ADI‐900‐011) and testosterone (#ADI‐900‐065) from Enzo Life 218 Sciences (https://www.enzolifesciences.com) and for T3 from Arbor Assays (#K056‐H1, 219 https://www.arborassays.com) were used to measure the concentrations of faecal glucocorticoid (fGC), progestin, androgen and thyroid metabolites in each sample, respectively. Assay validations and QA/QC protocols are described in Lemos, Olsen, et al. (2020) and Pirotta, Fernandez Ajó, et al. (2023).

### 2.2 Model development

**FIGURE 1:**
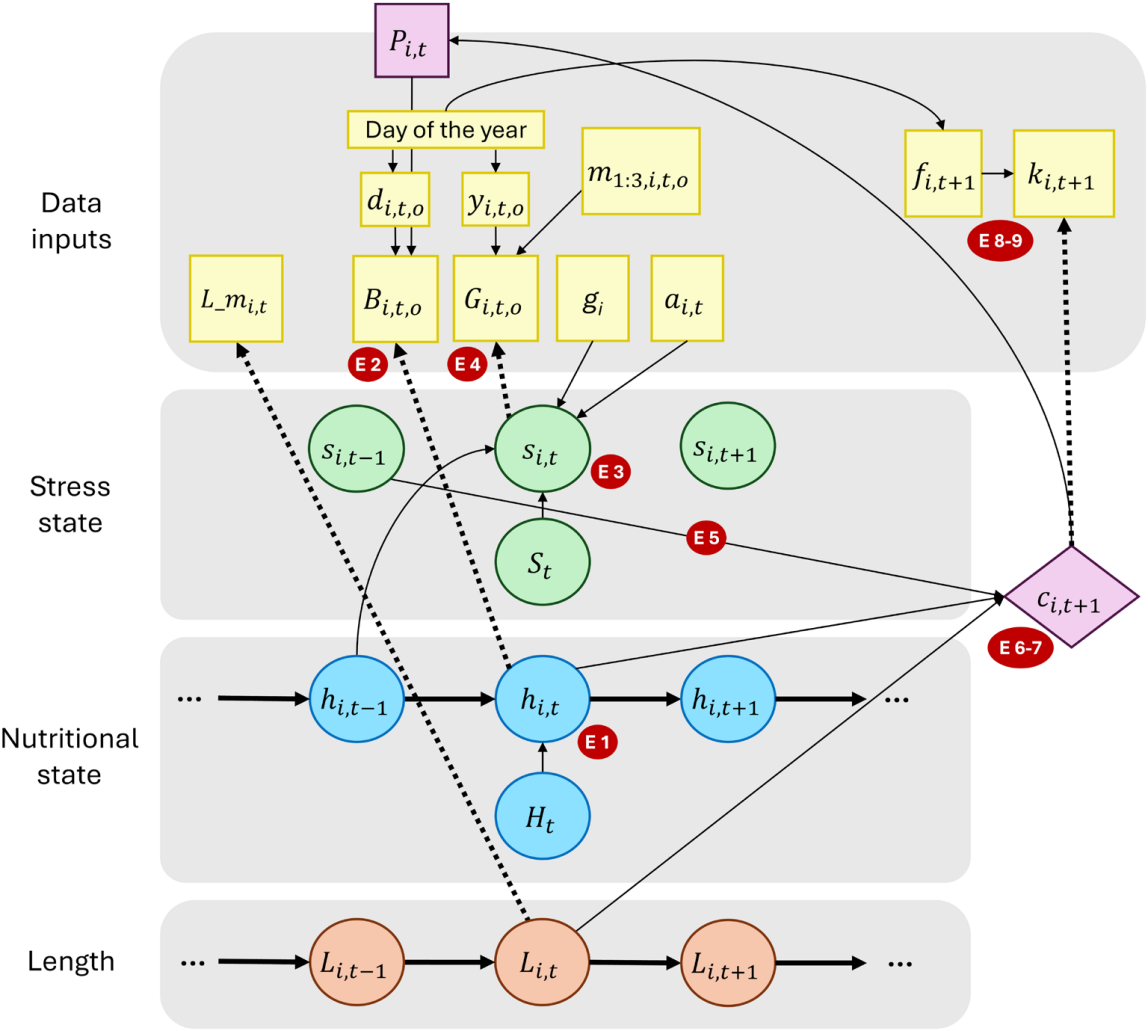
Schematic representation of the state–space model. Latent states are represented as circles, vital rates as diamonds and data streams as squares (note that pregnancy status is considered as a partial data stream, because it is informed partly by observations of calves and partly by estimated events that were missed). Model processes are represented for individual *i* at time step *t*; the corresponding equation numbers are reported in the small red circles. The time series of nutritional state, $h_{i,t}$, depends on the population mean for a given year, $H_{t}$. This state is informed by the body area index, $B_{i,t,o}$, which also depends on when it is measured relative to the end of the field season ($d_{i,t,o}$) and pregnancy status ($P_{i,t}$). The stress state, $s_{i,t}$, depends on the population mean for a given year ($S_{t}$), nutritional state in the previous time step ($h_{i,t–1}$), sex ($g_{i}$) and age ($a_{i,t}$) from the model by Pirotta, Bierlich, et al., (2024) and is informed by faecal sampling. The latter is represented here as the expected faecal glucocorticoid concentration ($G_{i,t,o}$), given day of sampling ($y_{i,t,o}$) and the concentrations of the other three hormone metabolites ($m_{1:3,i,t,o}$). Individual length, $L_{i,t}$, is informed by the estimates (with uncertainty) from the model by Pirotta, Bierlich, et al. (2024), ${\text{L\_m}}_{i,t}$. Nutritional state, length and the residual of the stress state (not represented here, for simplicity) affect whether a female gives birth to a calf ($c_{i,t+1}$), which, in turn, determines pregnancy status the year before ($P_{i,t}$). Calves can be observed in the study area ($k_{i,t+1}$), which depends on the day a female is first sighted ($f_{i,t+1}$).

We developed a Bayesian state–space model for the health and calving probability of individual PCFG gray whales (Figure 1), building on the approach in Pirotta, Schick, et al. (2023) and Schick et al. (2013). In the model, individual health is taken as the combination of two processes (nutritional and stress states), each associated with a data stream that informs the underlying, unobserved state. The time step for the model is 1 year; nutritional state is assumed to represent an individual's bioenergetic condition at the end of the feeding season in October of each year (before whales migrate to their wintering grounds), whereas the stress state is conceptualised as the average state of hypothalamic–pituitary–adrenal axis activation over the feeding season (Sapolsky, 2002). Calving probability is modelled for sexually mature females (i.e. ≥8 years; Rice & Wolman, 1971) in years when they were available to calve (i.e. not pregnant), and a successful reproductive event is defined as a female giving birth to a calf. Note that we modelled this unobserved event separately from the observation process that led to the sighting of a female with a calf in the study area (see description of the observation model below).

The time series of an individual's latent nutritional state, $h_{i,t}$, is modelled as:

(1) $$h_{i,t}=H_{t}+\beta_{h}\;h_{i,t-1}^{*}+\varepsilon_{i,t}$$

where $H_{t}$ is a normally distributed population‐level random effect of year, with mean $\lambda_{h}$ and standard deviation $\chi_{h}$, $\beta_{h}$ is the effect of nutritional state in the previous time step (which was standardised by subtracting the mean and dividing by the standard deviation to facilitate model convergence, indicated by ‘*’), and $\varepsilon_{i,t}$ is a normally distributed error with mean 0 and standard deviation $\sigma_{h}$.

An individual's latent nutritional state is unitless, and it affects the BAI measurements. BAI is therefore an emergent property, which is also influenced by other processes. Specifically, given that we take $h_{i,t}$ to reflect the state at the end of the feeding season, we correct BAI measurements to account for increasing body condition over the summer months. Moreover, we expect a pregnant female to have a larger BAI due to the effect of the foetus on her body shape (i.e. irrespective of her energy reserves, Fernandez Ajó et al., 2023). As a result, we modelled the expected BAI of individual *i*, at observation time *o* in year *t*, as:

(2) $$\begin{array}{c} B_{i,t,o}=h_{i,t}+\delta_{1}\;d_{i,t,o}+\delta_{2}\;P_{i,t}+\omega_{i,t,o} \end{array}$$

where $d_{i,t,o}$ is the number of days between measurement date and the end of the field season (15 October), $P_{i,t}$ is a binary variable indicating whether the individual was pregnant in that year, $\delta_{1}$ and $\delta_{2}$ are the respective coefficients of the two effects, and $\omega_{i,t,o}$ is a normally distributed error with mean 0 and standard deviation $\nu_{h}$. We incorporate the uncertainty associated with BAI measurements (Bierlich et al., 2021) by modelling posterior mean BAI estimates from the extension of the model in Pirotta, Bierlich, et al. (2024) (Supporting information) as normally distributed around $B_{i,t,o}$, with standard deviation also from the extended model.

Based on Pirotta, Fernandez Ajó, et al. (2023), we hypothesise that an individual's latent stress state in a given year, $s_{i,t}$, is affected by its standardised nutritional state at the end of the previous feeding season ($h_{i,t-1}^{*}$), standardised age ($a_{i,t}^{*}$) and sex ($g_{i}$):

(3) $$\begin{array}{c} s_{i,t}=S_{t}+\beta_{s,1}\;h_{i,t-1}^{*}+\beta_{s,2}\;a_{i,t}^{*}+\beta_{s,3}\;g_{i}+\upsilon_{i,t} \end{array}$$

where $S_{t}$ is a normally distributed population‐level random effect of year, with mean $\lambda_{s}$ and standard deviation $\chi_{s}$, $\beta_{s,1:3}$ are the coefficients for the effects of the covariates, and $\upsilon_{i,t}$ is a normally distributed error with mean 0 and standard deviation $\sigma_{s}$. Based on preliminary analyses, we do not include an autoregressive term for this state process (i.e. $s_{i,t-1}$). We use posterior age estimates from Pirotta, Bierlich, et al. (2024) to incorporate age uncertainty, while imposing a constraint based on an individual's known minimum age ($\min_{i,1}$), that is 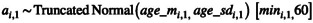. When sex was unknown, it was imputed in the Bayesian model from a Bernoulli prior, assuming a 1:1 sex ratio.

An individual's latent stress state affects the concentration of glucocorticoid metabolites in faecal samples (fGC). Though fGC data are context‐dependent and not synonymous with “stress” (MacDougall‐Shackleton et al., 2019), data across species consistently indicate that they are a useful proxy of hypothalamic–pituitary–adrenal axis activation (Dantzer et al., 2014). Therefore, we assume that whales experiencing prolonged physiological stress will, on average, have elevated fGC concentrations (e.g. Dickens & Romero, 2013; Pirotta, Fernandez Ajó, et al., 2023). Further, important context is provided via the known associations of fGC with the concentrations of other hormone metabolites (progestin, androgen and thyroid hormone, log‐transformed and symbolised as $m_{1}$, $m_{2}$ and $m_{3}$, respectively) and the day of the year when an individual was sampled ($y_{i,t,o}$) (Pirotta, Fernandez Ajó, et al., 2023). Therefore, we model the expected glucocorticoid concentration given these covariates for individual *i* in year *t* in faecal sample *o* ($G_{i,t,o}$) as:

(4) $$\begin{array}{c} G_{i,t,o}=s_{i,t}+\zeta_{1}\;m_{1,i,t,o}+\zeta_{2}\;m_{2,i,t,o}+\zeta_{3}\;m_{3,i,t,o}+\zeta_{4}\;y_{i,t,o}. \end{array}$$

When the concentrations of the other hormones were below the limit of detection of the assays, they were imputed in the Bayesian model from uniform distributions; the limit of detection was used as the upper boundary, except for T3 in 2016, when this hormone was not measured and for which the maximum observed concentration was used. The expected concentration $G_{i,t,o}$ is then exponentiated (Pirotta, Fernandez Ajó, et al., 2023) and acts as the mean of a Normal distribution (with standard deviation $\nu_{s}$), from which the faecal glucocorticoid measurements emerge, that is $fGC_{i,t,o}∼\mathrm{Normal}\left(\exp\left(G_{i,t,o}\right),\;\nu_{s}\right)$.

A female's calving probability in year *t*, $\varphi_{i,t}$, is modelled as a function of her standardised nutritional state (i.e. subtracting the mean and dividing by the standard deviation) in the previous year ($h_{i,t-1}^{*}$) and length in the previous year, which was cubed and standardised following Pirotta, Tyack, et al. (2024) ($L_{i,t-1}^{3*}$). We use the posterior body length estimates from Pirotta, Bierlich, et al. (2024) to incorporate uncertainty in length, that is $L_{i,t}∼\mathrm{Normal}\left({\text{L\_m}}_{i,t},\;{\text{L\_sd}}_{i,t}\right)$ . We also model the effect of the residual stress state 2 years prior on calving probability ($r_{i,t-2}$; see Supporting Information for a discussion of the selection of this time lag). We use the residual because we hypothesise that calving probability is associated with the deviation from the population's and demographic class's stress baselines, and to avoid double‐counting the effect of nutritional state (which is also modelled to affect the stress state), and we define it as:

(5) $$\begin{array}{c} r_{i,t}=s_{t}-\left(\lambda_{s}+\beta_{s,1}\;h_{i,t-1}^{*}+\beta_{s,2}\;a_{i,t}^{*}+\beta_{s,3}\;g_{i}\right). \end{array}$$

A female's calving probability was modelled starting at sexual maturity, with some individuals entering the time series as sexually mature, as:

(6) $$\begin{array}{c} \text{logit}\left(\varphi_{i,t}\right)=\gamma_{1}+\gamma_{2}\;h_{i,t-1}^{*}+\gamma_{3}\;L_{i,t-1}^{3*}+\gamma_{4}\;r_{i,t-2} \end{array}$$

which determines whether a female calved or not in year *t*:

(7) $$\begin{array}{c} c_{i,t}∼\text{Bernoulli}\left(\varphi_{i,t}\;\left(1-P_{i,t}\right)\right) \end{array}$$

The minimum reported inter‐calf interval for gray whales is 2 years (Jones, 1990). Therefore, we include the term $\left(1-P_{i,t}\right)$ because a female cannot give birth in a year when she is pregnant, and we assume that longer inter‐calf intervals would emerge from suboptimal conditions affecting $\varphi_{i,t}$ (e.g. poor nutritional status). If $c_{i,t}=1$, a female must have been pregnant the previous year, that is $P_{i,t-1}=c_{i,t}$. In the last time step, the pregnancy status of a female is estimated from a Bernoulli distribution with probability 0.05, which is the mean proportion of known pregnant females per year in 2016–2022.

We include an observation model for the probability of observing a female's calf in our surveys, which depends on the day of first sighting of that female, $f_{i,t}$, because females have an increasing probability of weaning their calf as the season progresses (Calambokidis & Perez, 2017):

(8) $$\begin{array}{c} \text{logit}\left(p_{i,t}\right)=\eta_{1}+\eta_{2}\;f_{i,t} \end{array}$$

This probability underpins calf observations ($k_{i,t}$), which are also set to 0 if a female was never encountered in a given year ($e_{i,t}=0$):

(9) $$\begin{array}{c} k_{i,t}∼\text{Bernoulli}\left(c_{i,t}\;p_{i,t}\;e_{i,t}\right) \end{array}$$

Note that if a calf was seen by another research group but not in our study area, $c_{i,t}=1$ and $k_{i,t}=0$.

This model structure was chosen to balance our understanding of the mechanistic processes underpinning the system (Figure S4), the research questions of interest and data availability. The model was fitted in a Bayesian framework using Markov Chain Monte Carlo algorithms in package NIMBLE (de Valpine et al., 2017) for R (R Core Team, 2023). Details of parameter priors and constraints, fitting and model diagnostics are reported in the Supporting Information.

All methods were carried out in accordance with relevant guidelines and regulations. This project was approved by the Oregon State University Institutional Animal Care and Use Committee (IACUC‐ 2019‐0008). Analysis of the data was also approved by the Animal Welfare and Ethics Committee (AWEC) of the University of St Andrews (UK). All gray whale data collection was carried out under a research permit from NOAA/NMFS (#16011 and #21678, issued to John Calambokidis). Drone operations were conducted by a Federal Aviation Authority (FAA) certified private pilot with a Part107 licence and under a yearly Certificate of Authorization.

## 3 RESULTS

**FIGURE 2:**
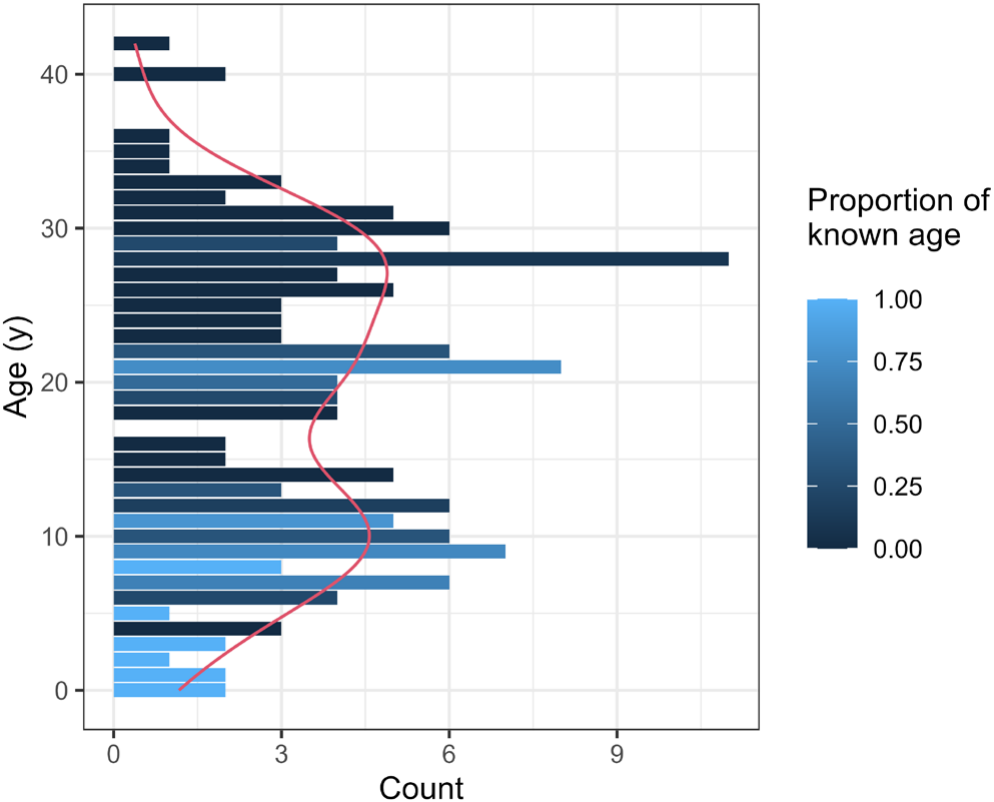
Age structure diagram for 139 PCFG gray whales in our sample. Each bar represents the number of individuals of a given age in 2023, with the colour indicating the proportion of individuals of that age for which age is known (vs. estimated from a minimum estimate following Pirotta, Bierlich, et al., 2024). The red line reports a smooth kernel density estimate of the distribution.

The final dataset included 139 individual gray whales (46 males, 55 females and 38 individuals of unknown sex, representing approximately 65% of the PCFG; Harris et al., 2022) whose nutritional and stress states were modelled for up to 8 time steps (2016–2023, unless the individual was born during the study period). Age was known for 29% of individuals in the sample. BAI was measured 665 times (Figure S1), averaging 5 times per individual (range: 1–36). fGC concentration was measured 337 times (Figure S2) in a subset of 84 individuals, 4 times per individual on average (range: 1–35). Fifty‐four females of known or estimated age ≥8 years were included in the calving model. In 2016–2023, we observed 15 calves in our study area, with an additional 7 calves reported by other research groups (2 of the corresponding mothers were observed in our study area without a calf later in the season), from a total of 17 different females (Figure S3). The apparent age structure of the population in 2023 highlighted a proportionally small number of individuals ≤5 years old (Figure 2).

The chains mixed and converged appropriately according to all diagnostics (posterior results in Supporting Information). Parameter estimates are reported as posterior medians, followed by the 95% credible interval (CI) in square brackets.

**FIGURE 3:**
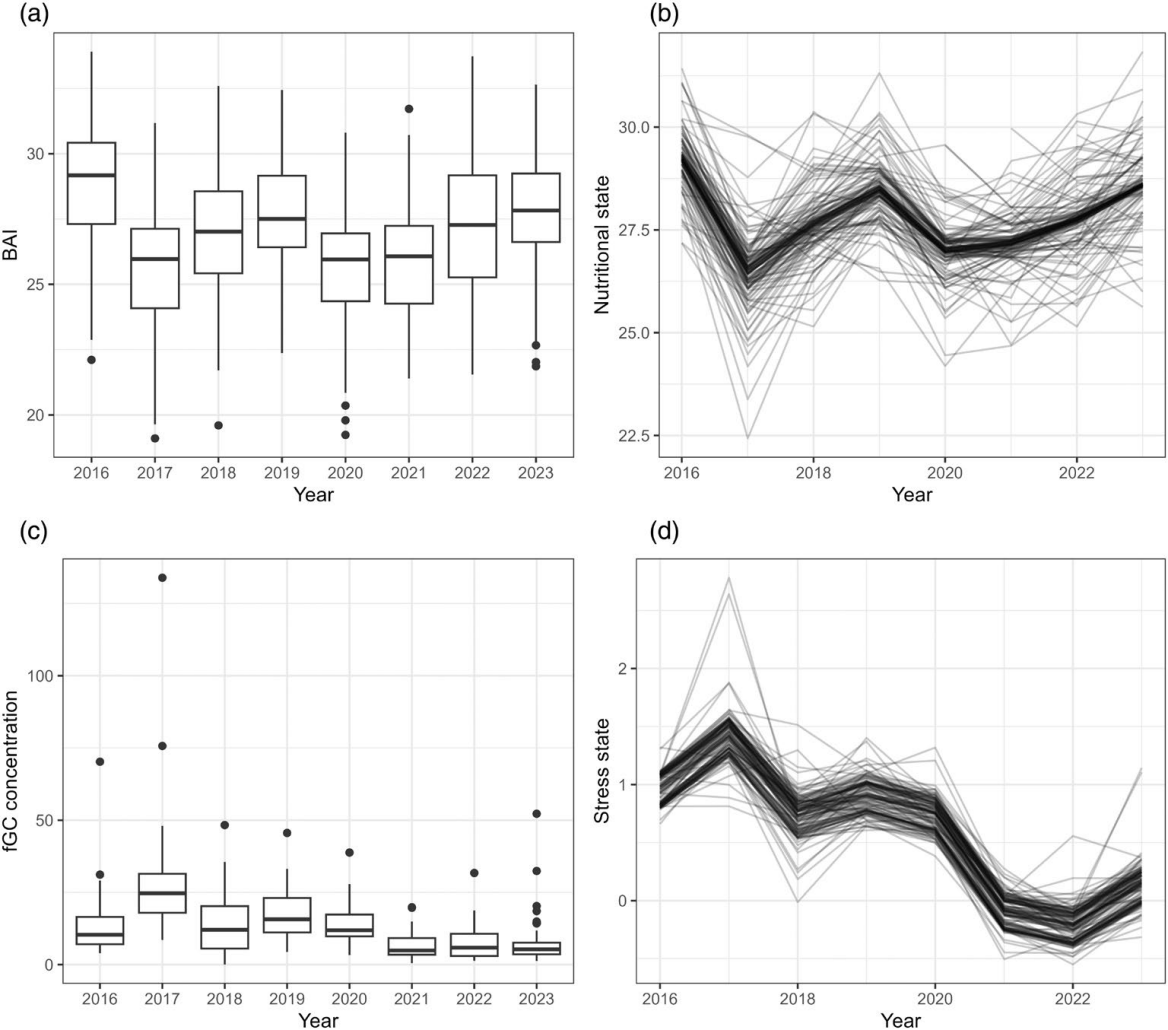
Distribution of observed body area index (BAI; a) and faecal glucocorticoid concentration (fGC; c) measurements in each year, which underpin the estimated nutritional (b) and stress (d) states. In (b and d), each line represents the state trajectory for one individual.

The population nutritional state at the end of the feeding season varied interannually, following the patterns in measured BAI, although individual trajectories deviated from the mean trend (Figure 3). The estimated stress state behaved similarly, but the deviation from the population mean state was lower due to the smaller sample of fGC observations (Figure 3). Nutritional state at the end of a given feeding season was associated with nutritional state at the end of the previous season ($\beta_{h}$ = 0.40 [0.14–0.61]). Females had a higher stress state on average ($\beta_{s,3}$ = 0.26 [0.03–0.49]). Nutritional state in the previous season was negatively related with stress state, but the 95% CI showed a small overlap with 0 (specifically, there is an 88% chance that being in a better nutritional state results in a lower stress state; $\beta_{s,1}$ = −0.09 [−0.22–0.06]).

**FIGURE 4:**
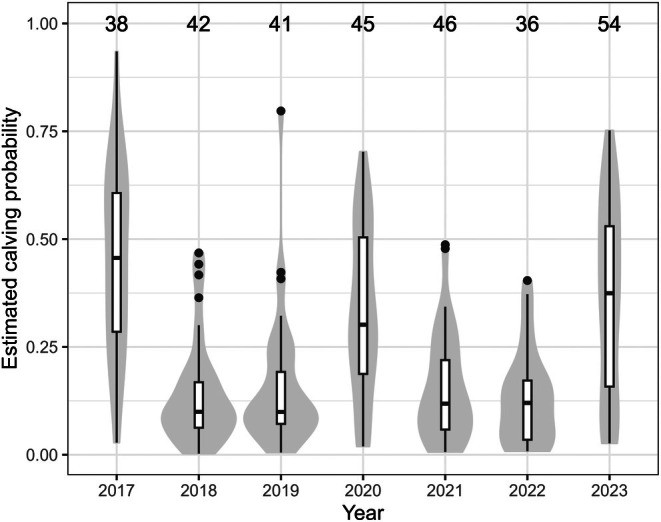
Distribution of estimated calving probabilities across females included in the model. The number of non‐pregnant, mature females available to reproduce in each year is reported at the top.

Mean calving probability was highly variable among individuals and over time, with the median varying between 0.10 [0.02–0.42] in 2019 and 0.46 [0.10–0.78] in 2017 (Figure 4). Mean estimated calving probabilities were partially influenced by model formulation, particularly at the extremes (Supporting Information).

**FIGURE 5:**
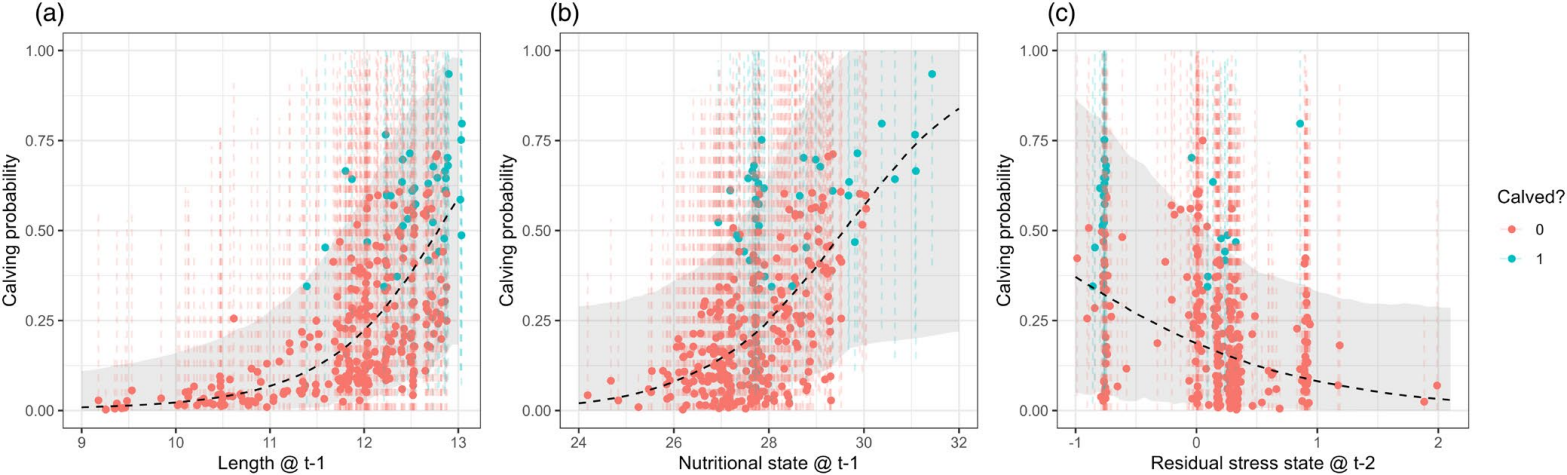
Individual length (a), nutritional state (b) and residual stress state (c) plotted against the estimated calving probability (the dots and dashed segments indicate the posterior medians and 95% CI), and coloured by whether the model estimated an individual to have calved or not at a given reproductive opportunity. The black dashed lines and grey ribbons represent the estimated relationship (median and 95% CI) between each covariate and calving probability when the other two covariates are set to their mean.

**FIGURE 6:**
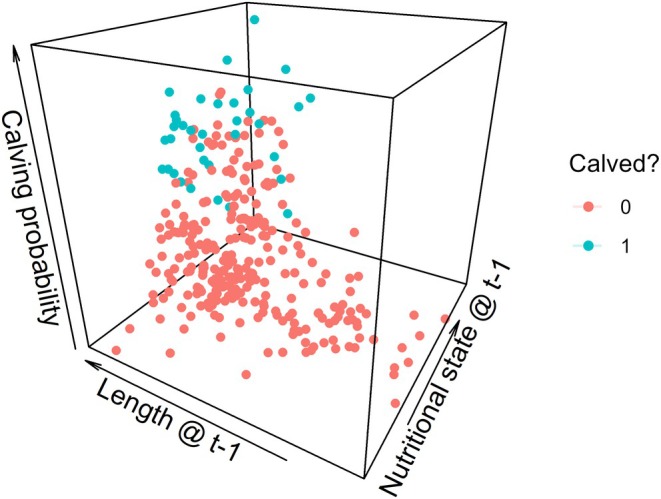
Combined relationship of length and nutritional state in the previous time step with calving probability, coloured by whether the model estimated an individual to have calved or not at a given reproductive opportunity.

All three covariates in the calving model showed some association with calving probability (Figure 5). The cube of body length in the previous time step had a positive effect ($\gamma_{3}$ = 1.36 [0.29–2.64]). As an example, calving probability was 0.01 [0–0.13] for a 9.5‐m long female and 0.59 [0.18–0.98] for a 13‐m long female; no female ≤11.5 m was estimated to have reproduced. The effect of nutritional state in the previous time step was positive but showed a small overlap with 0 ($\gamma_{2}$ = 0.80 [−0.11–2.38]; 95% of the posterior distribution was ≥0). Calving probability varied from 0.02 [0–0.29] to 0.84 [0.22–1.00] for nutritional state $h_{i,t}$ varying from 24 to 32, with no female was estimated to have reproduced below ~27. Combined, the effects of length and nutritional state highlighted a range of morphometric features associated with increased calving probability (Figure 6). The median effect of the stress state residual two time steps prior to a reproductive opportunity was negative, but the 95% CI showed some overlap with 0 ($\gamma_{4}$ = −0.97 [−2.48–0.39]; 92% of the posterior distribution was ≤0), with calving probability varying from 0.37 [0.05–0.86] to 0.03 [0–0.29] over the range of stress residual values (Figure 5c). The prior distributions for these parameters had some influence on the strength of the estimated relationships (Supporting Information). Overall, the calving model correctly predicted 87% of positive observations and 90% of negative observations, with an area under the receiver operating characteristic curve of 0.96, indicating good fit to the calving data.

The observation model for nutritional state identified the effect of day of the year (negative for increasing time from the end of the season; $\delta_{1}$ = −0.016 [−0.020 to −0.012]) and pregnancy status (mostly positive; $\delta_{2}$ = 0.63 [−0.35 to 1.95]) on measured BAI values. For fGC, the observation model included a positive effect of the log‐transformed concentrations of the other hormones ($\zeta_{1}$ = 0.30 [0.21–0.40], $\zeta_{2}$ = 0.18 [0.09–0.26] and $\zeta_{3}$ = 0.23 [0.18–0.30]) and a small negative effect of day of the year ($\zeta_{4}$ = −0.003 [−0.005 to −0.001]). Finally, calving events were estimated to be observed in our study area with an average probability of 0.62 ($\eta_{1}$ = 0.48 [−0.75–2.13]), which declined as the season progressed ($\eta_{2}$ = −1.80 [−3.86–0.16]). It should be noted that different model formulations resulted in some variation in coefficient estimates and in the number of calves estimated to have been missed each year (Supporting Information). In the model presented here, it was estimated that 19 calving events were missed in 2016–2023.

## 4 DISCUSSION

Using a detailed 8‐year dataset of repeated individual sightings, morphological measurements, faecal sampling and offspring observations, we developed a Bayesian state–space model for the health indicators and calving probability of individuals in a long‐lived population. Our results indicate that the probability of a female PCFG gray whale calving in any given year emerges from the combined effect of her structural body size and the amount of energy reserves available to invest in a reproductive attempt. Specifically, there appears to be a range of body length and body condition values that are required to support a successful reproductive event, with no females estimated to have produced a calf if they were ≤11.5 m long or at a nutritional state <27 (just below the median estimated state across individuals and time steps of 27.7). Stress state, defined here as average hypothalamic–pituitary–adrenal axis activity (observed via fGC data), was also negatively correlated with calving probability. This association had large uncertainty but was strongest at a 2‐year lag, suggesting that the physiological stress experienced by a female may influence her ability or decision to become pregnant.

The minimum observed length at calving could act as a proxy of age at maturity, and/or a minimum length required to reproduce (Stearns, 1992) (see Supporting Information for an exploration of the potential residual effects of age after a female has reached asymptotic body length). We used the cube of body length because we expected it to represent structural size (Pirotta, Tyack, et al., 2024), affecting the maximum amount of reserves a female can carry (Millar & Hickling, 1990). Our results support this hypothesis, and align with evidence that body size is associated with reproductive success in a range of mammal species (e.g. Post et al., 1997; Rode et al., 2010; Wauters et al., 2007). While body size relates to the absolute amount of energy stores (Adamczak et al., 2023), female relative body condition in any given year (as represented by BAI) is also expected to be an important driver of reproductive outcome. Capital breeders, such as gray whales, predominantly rely on energy stored during the feeding season to support the costs of late gestation and early lactation (Stephens et al., 2009) and Beltran et al. (2023) hypothesised the existence of physiological tipping points (that is, a step‐like relationship with energetic status) that determine whether a female reproduces. The estimated relationship between nutritional state at the end of a feeding season and the observation of a female with a calf the following year reinforces the importance of a successful feeding season in supporting reproduction. The mother's body condition could also affect foetus growth and size at birth (Christiansen et al., 2014), which in turn could be associated with calf survival probability (as observed in pinnipeds, e.g. Harding et al., 2005); a calf born in poor condition might then die before we observe it on the feeding grounds.

Our results highlight that an individual's life history performance is not solely dependent on its bioenergetic status; there are other aspects of health that contribute to fecundity, such as physiological stress. Here, our goal was to capture the deviation of an individual's mean stress state during a feeding season from baseline population levels, given its demographic characteristics and other contextual factors associated with fGC (Dickens & Romero, 2013). We found a weak negative correlation between residual stress state and calving probability at a two‐year lag, which aligns with previous work on the role of cortisol in modulating implantation in northern elephant seals (*Mirounga angustirostris*; Sperou, 2020), as well as the general evidence that stress is associated with suppressed reproduction (e.g. Rivier & Rivest, 1991; Vitousek et al., 2018). Our study thus presents some empirical evidence that the hypothalamic–pituitary–adrenal axis, and by extension the physiological stress response, may also affect reproduction in baleen whales.

These results have implications for understanding the status of the PCFG. Their asymptotic body length has decreased over the last two decades (Pirotta, Bierlich, et al., 2024), with maximum length estimated to have dropped from 12.55 m [12.31–12.86] before 2000 to 10.99 m [10.23–11.74] for females born in 2020. According to our model, this could correspond to a change in asymptotic calving probability from 0.41 [0.09–0.86] to 0.07 [0.00–0.27] (notwithstanding the interplay between age and size that may affect the estimated relationship with calving probability). Because PCFG whales are in poorer body condition than Eastern North Pacific gray whales (Torres et al., 2022), this could also contribute to reduced calving probability; specifically, given the median BAI of PCFG and Eastern North Pacific whales (26.5 and 29.1, respectively; Torres et al., 2022), our model estimates a possible difference in calving probability of 0.11 [0.00–0.39] versus 0.41 [0.10–0.90].

Pirotta, Fernandez Ajó, et al. (2023) assessed the association between exposure to anthropogenic stressors (vessels and noise) and short‐term (~24 h) changes in fGC concentrations. Interpreting their results in the context of the relationship with stress residual in our year‐level model is complicated by the mismatch in time scale, as well as by the subtle and context‐dependent nature of detected physiological responses. Correlations between stressor exposure and fGC tended to be less apparent in females, although females showed higher fGC concentrations on average (Pirotta, Fernandez Ajó, et al., 2023, in line with our yearly results); therefore, females could be more stressed on average, potentially limiting their scope to respond to short‐term disturbance. Alternatively, they could have higher fGC baselines or seasonally down‐modulate their sensitivity to stress while engaged in reproduction (Wingfield et al., 1995). Our results suggest elevated fGCs may affect the initiation of breeding efforts, but it remains unclear whether this is attributable to specific stressors.

The model estimated a substantial number of missed calving events, which could correspond to early foetus/calf deaths, calves missed in our surveys, or calves weaned prior to observation with their mother (Calambokidis & Perez, 2017; Fernandez Ajó et al., 2023). Importantly, the number of missed calving events was dependent on model formulation: the short time series, combined with the observation model for calving, means that estimated calving probability is affected by model structure and assumptions. This suggests some caution in the interpretation of the results; our observation model may not be capturing all variables affecting calf detection, and additional health state variables driving calving probability may also be missing. Any missing state variable may confound the estimated relationships if these additional state processes interact with the state variables that were included. However, the apparent distribution of ages in the population suggests that the number of individuals in younger age classes is indeed smaller than one would expect from a growing or a stable population (Iannelli & Milner, 2017). The age distribution (Figure 2) suffers from age uncertainty for a large proportion of individuals, as well as from the missing individuals in our sample (our study includes ~65% of the PCFG). Nonetheless, even when interpretation is restricted to areas where age uncertainty is lower, the shape of the distribution highlights a potential lack in population growth, possibly driven by low calving probability, high perinatal/juvenile mortality or a combination of both. Therefore, although PCFG abundance is thought to be stable (Barlow et al., 2024; Harris et al., 2022), the few individuals of younger ages, together with the decreasing trend in asymptotic body length (Pirotta, Bierlich, et al., 2024), could constitute early warnings of an impending population decline, following the timeline proposed by Cerini et al. (2023). Whatever the initial motivations that led gray whales to use the separate PCFG summer range instead of foraging grounds in the Arctic and sub‐Arctic, this evidence could suggest that their habitat is potentially becoming an ecological trap (Torres et al., 2022) or that it cannot sustain many more individuals than it currently does. Yet, estimated calving probability was variable from year to year, suggesting gray whales may have some ability to respond plastically to, and buffer against, changing environmental conditions (Perryman et al., 2021). Therefore, a research priority is a concerted effort to expand the monitoring of calves' occurrence across the PCFG range, to reduce uncertainty around calving probabilities and number of missed births. More generally, these results illustrate the importance of individual‐based, long‐term studies where potential early warnings of population decline (e.g. morphology, young animal survival and reproductive rates; Cerini et al., 2023; Eberhardt, 2002) are monitored explicitly.

The development of a state–space model for gray whale health and calving probability represents one of the first attempts at modelling multiple health indicators (energy status, endocrine status and morphometric status) concurrently, and informing the underlying processes via an array of diverse data streams (National Academies, 2017; Tyack et al., 2022). While more health markers will be needed for a comprehensive representation of individuals' health (Schwacke et al., 2024), we provide an example of how multivariate data can be used in an integrated model to explore the causal pathways that connect various aspects of health to vital rates. Our work also demonstrates the applicability of the modelling framework beyond North Atlantic right whales, an endangered species that has been intensively monitored and for which this approach was originally conceived (Pirotta, Schick, et al., 2023; Schick et al., 2013). The adaptation of the model to a species with a shorter data collection period and a different conservation status has shifted the focus away from individual survival and onto calving probability, which is the vital rate that is anticipated to change first in a non‐declining population (Cerini et al., 2023; Eberhardt, 2002). The relatively infrequent use of state–space modelling to track latent individual traits and their relationships with fitness has potentially wide applications across marine and terrestrial taxa (e.g. New et al., 2014; Pigeon et al., 2022).

The short time series (8 years) meant model formulation strongly affected the results (Pirotta et al., 2022). For example, the assumption that individual nutritional and stress states are distributed around a population yearly mean drove the estimation of these states in years when individuals were not observed (as demonstrated by the tendency for individual states to follow the population‐level trends in Figure 3). Moreover, we included the effect of pregnancy on body shape in the observation model for nutritional state, but it is unclear how well the model is apportioning BAI to various components (see Supporting Information where we explore a restricted calculation of BAI). Pregnancy also induces other changes in a female's metabolism (e.g. increased fat deposition; Zeng et al., 2017) that could contribute to the estimated relationship between accumulated energy reserves and probability of giving birth, but these effects could not be teased apart using available data. Independent data on pregnancy status would also support discriminating the factors affecting the initiation of pregnancy from those that influence its successful completion. The prior distributions of model parameters had some influence on the strength of estimated relationships. These decisions were guided by a mechanistic understanding of the system, but could lead to erroneous results if those assumed mechanisms were incorrect (Pirotta et al., 2022). Data availability also limited the number of covariates included in the state processes and the feedback mechanisms among these processes. While we focused on testing specific hypotheses of interest, future theoretical work should further explore these mechanisms, which will ultimately support the development of robust empirical models (Pirotta et al., 2022).

The model also had to be modified to operate at a coarser temporal scale than for North Atlantic right whales, and the limited heterogeneity in life‐history performance in this non‐threatened population meant that the estimated connections between model components were more uncertain. However, the results demonstrate that it is feasible to develop these models in a time frame compatible with management and conservation requirements (i.e. before decades of data are available and before a population is declining; Tyack et al., 2022). We thus reinforce the conclusions of previous work suggesting that data on individual health and life‐history performance are critical (Keen et al., 2021; Pirotta et al., 2018; Pirotta, Schick, et al., 2023; Tyack et al., 2022). In particular, we argue that it was the intense effort in collecting high‐resolution, longitudinal data on individuals that made it possible to develop the model presented here. The more precise metric of body condition (BAI; Bierlich et al., 2021) was especially useful to capture the subtler changes that are likely to drive calving probability, as opposed to survival (Pirotta, Tyack, et al., 2024).

Advancing research on health and vital rate modelling can support assessments of the population‐level consequences of multiple stressors, which is a key goal for management and conservation across a range of ecological systems, research disciplines and regulatory jurisdictions (Tyack et al., 2022). The next step in this work will therefore be to incorporate the effects of stressors explicitly in the underlying state processes (i.e. nutritional state, stress state or length; Pirotta, Schick, et al., 2023). This will be complicated by the coarse temporal scale at which the model operates and the challenges of quantifying exposure rates robustly, especially given that our study area is a subset of the PCFG range. Alternatively, targeted analyses could estimate the effects of stressors on length, body condition and stress separately, to be then used in combination with our estimated relationships with calving probability in a simulation framework. Ultimately, mechanistically informed modelling approaches will be critical to assess the combined effects of stressors on individual health and vital rates, supporting the development of effective management scenarios to reduce stressor‐inducing activities and ensure the viability of wildlife populations.

## AUTHOR CONTRIBUTIONS

Enrico Pirotta, Leigh G. Torres and Leslie New conceived the study, with contributions from the other co‐authors; Enrico Pirotta developed and ran the analyses, with support from Leslie New; Leigh G. Torres, Alejandro Fernandez Ajó, K.C. Bierlich, Clara N. Bird and Lisa Hildebrand collected and obtained permissions for use of the data; K.C. Bierlich and Clara N. Bird processed the photogrammetry data; Alejandro Fernandez Ajó processed the hormone data, with guidance from C. Loren Buck and Kathleen E. Hunt; Leigh G. Torres and Lisa Hildebrand coordinated the data sharing with other research groups; Lisa Hildebrand provided guidance on individual identification and processing of the calving data; John Calambokidis oversaw the long‐term photo‐ID and sightings database of PCFG gray whales; Enrico Pirotta led the writing of the manuscript. All authors contributed critically to the drafts and gave approval for publication.

## CONFLICT OF INTEREST STATEMENT

The authors declare that they have no conflict of interest.

## STATEMENT ON INCLUSION

Our study involved scientists from different countries, including the country where the study was carried out. Whenever relevant, literature published by scientists from the region was cited. We discussed the rationale of the study with several local research groups and other stakeholders (e.g. whale watching companies, Native American tribe), some of whom have agreed to contribute their information on observed individuals and calves.

## Supporting information


**Figure S1.** Temporal distribution of Body Area Index (BAI) estimates over the 8 years of the study period.
**Figure S2.** Temporal distribution of faecal glucocorticoids (fGC) measurements over the 8 years of the study period.
**Figure S3.** Temporal distribution of calf observations over the 8 years of the study period.
**Figure S4.** Gray whale model represented as a directed acyclic graph.
**Figure S5.** Residual stress state in the year prior to a reproductive opportunity, plotted against the estimated calving probability.
**Figure S6.** Distribution of estimated calving probabilities across females in the model without the stress state process (i.e. only including the effects of nutritional state and body length on calving probability).
**Figure S7.** A drone image of a gray whale, highlighting the region of the body used to calculate the standard BAI (i.e. the head‐tail range, including perpendicular widths in 5% increments of total length between 20% and 70%) and the restricted 20%–40% range.
**Table S1.** Prior distribution and posterior mean, standard deviation (SD), and 2.5th, 50th (median) and 97.5th quantiles of model parameters.

## Data Availability

The data and code to run the analyses are stored on the Open Science Framework repository (https://osf.io/df9yq; Pirotta et al., 2025).
