# Supplementary material for: Body size, nutritional state and endocrine state are associated with calving probability in a long‐lived marine species
Source: J Anim Ecol. 2025 Jun 12;94(7):1422–34. doi: 10.1111/1365-2656.70068 (PMC12214453; doi:10.1111/1365-2656.70068)
Supplement: Supplementary file 1 — Figure S1. Temporal distribution of Body Area Index (BAI) estimates over the 8 years of the study period. Figure S2. Temporal distribution of faecal glucocorticoids (fGC) measurements over the 8 years of the study period. Figure S3. Temporal distribution of calf observations over the 8 years of the study period. Figure S4. Gray whale model represented as a directed acyclic graph. Figure S5. Residual stress state in the year prior to a reproductive opportunity, plotted against the estimated calving probability. Figure S6. Distribution of estimated calving probabilities across females in the model without the stress state process (i.e. only including the effects of nutritional state and body length on calving probability). Figure S7. A drone image of a gray whale, highlighting the region of the body used to calculate the standard BAI (i.e. the head‐tail range, including perpendicular widths in 5% increments of total length between 20% and 70%) and the restricted 20%–40% range. Table S1. Prior distribution and posterior mean, standard deviation (SD), and 2.5th, 50th (median) and 97.5th quantiles of model parameters. [file JANE-94-1422-s001.docx]

**Supporting information**

***Table of contents***

[Temporal distribution of observations 2](#_Toc193104112)

[Directed acyclic graph representation of the model 5](#_Toc193104113)

[Prior distributions and posterior estimates of all model parameters 6](#_Toc193104114)

[Model fitting and diagnostics 9](#_Toc193104115)

[Extension of the growth model by Pirotta et al. (2024) to estimate body area index (BAI) 10](#_Toc193104116)

[Temporal lag between the residual of the stress state and calving probability 11](#_Toc193104117)

[Alternative model formulation excluding the stress state process 12](#_Toc193104118)

[Investigating the sensitivity of the results to the priors for the calving probability model 13](#_Toc193104119)

[Using a restricted estimate of body area index (BAI) 14](#_Toc193104120)

[Investigating the effect of age on calving probability 15](#_Toc193104121)

[References 16](#_Toc193104122)

Temporal distribution of observations


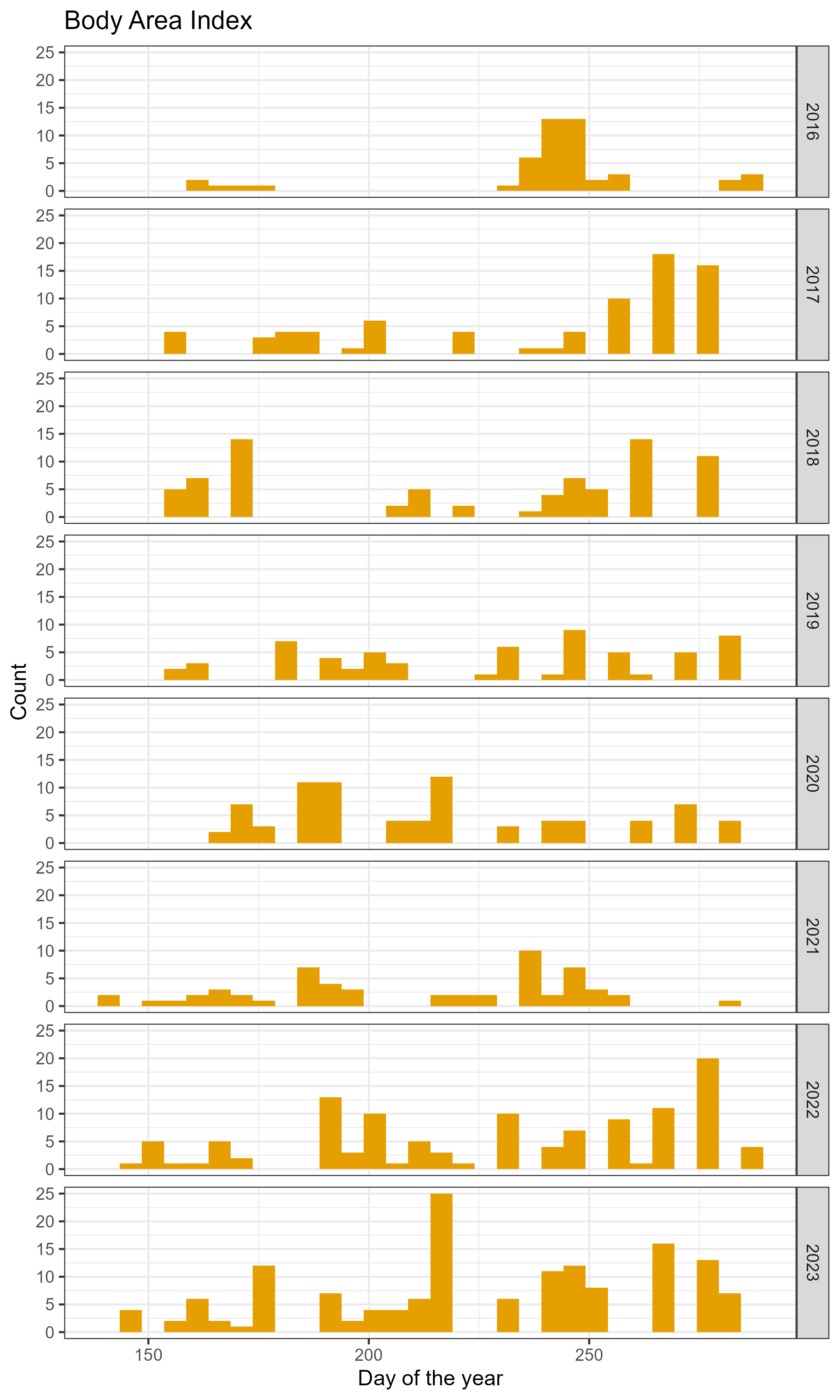


*Figure S1. Temporal distribution of Body Area Index (BAI) estimates over the eight years of the study period.*

***
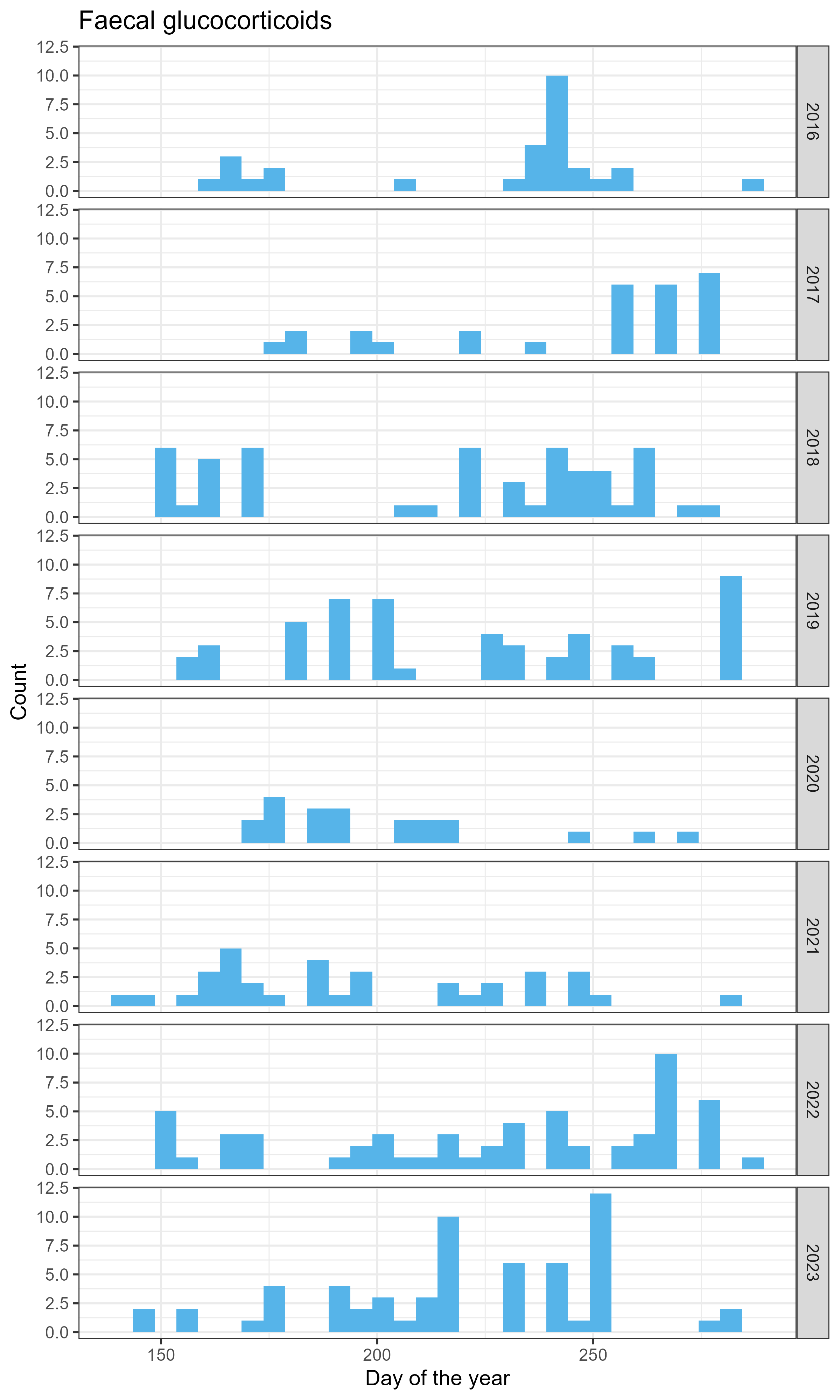
***

*Figure S2. Temporal distribution of faecal glucocorticoids (fGC) measurements over the eight years of the study period.*

***
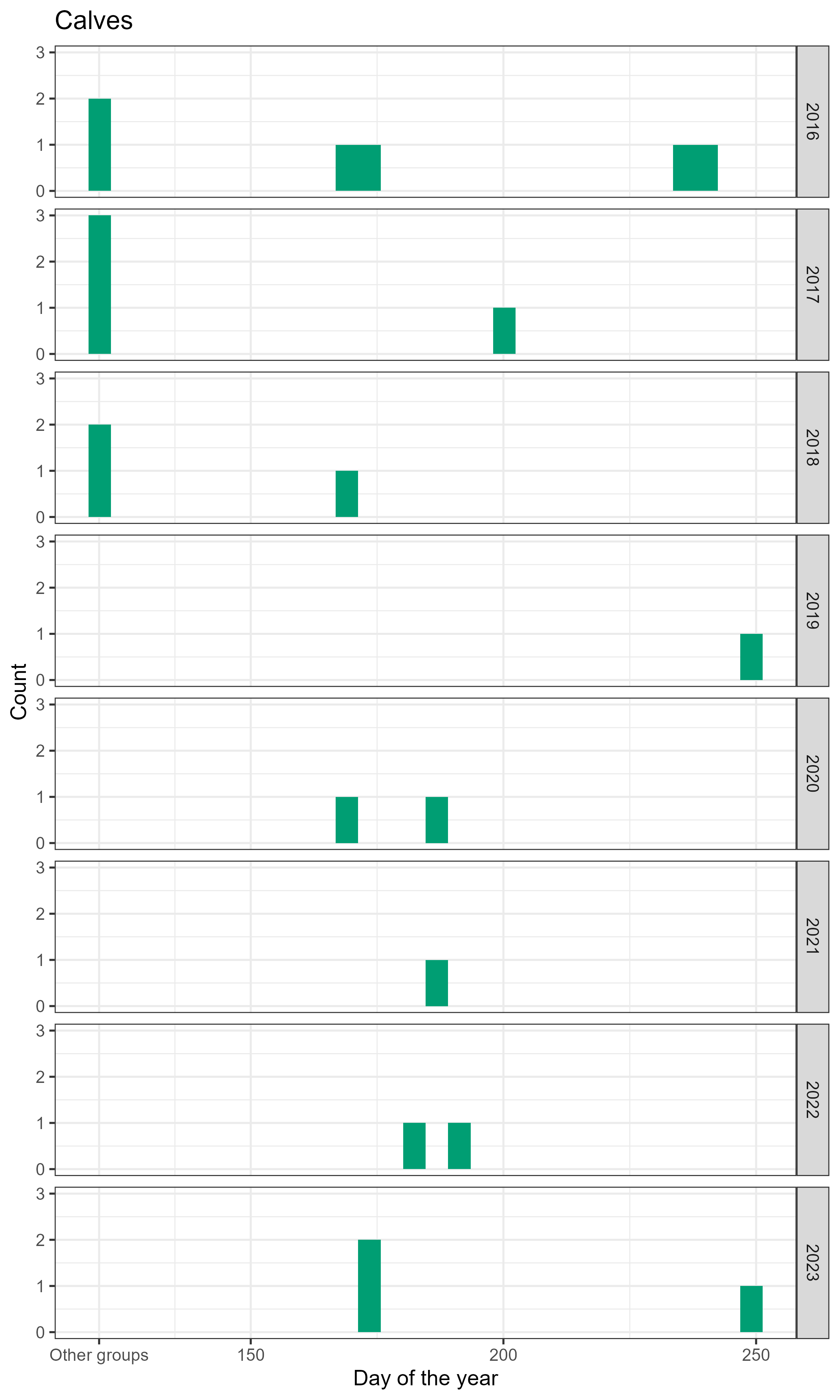
***

*Figure S3. Temporal distribution of calf observations over the eight years of the study period. Note that observations of calves by other research groups outside our study area are plotted separately on the left of each panel.*

Directed acyclic graph representation of the model

***
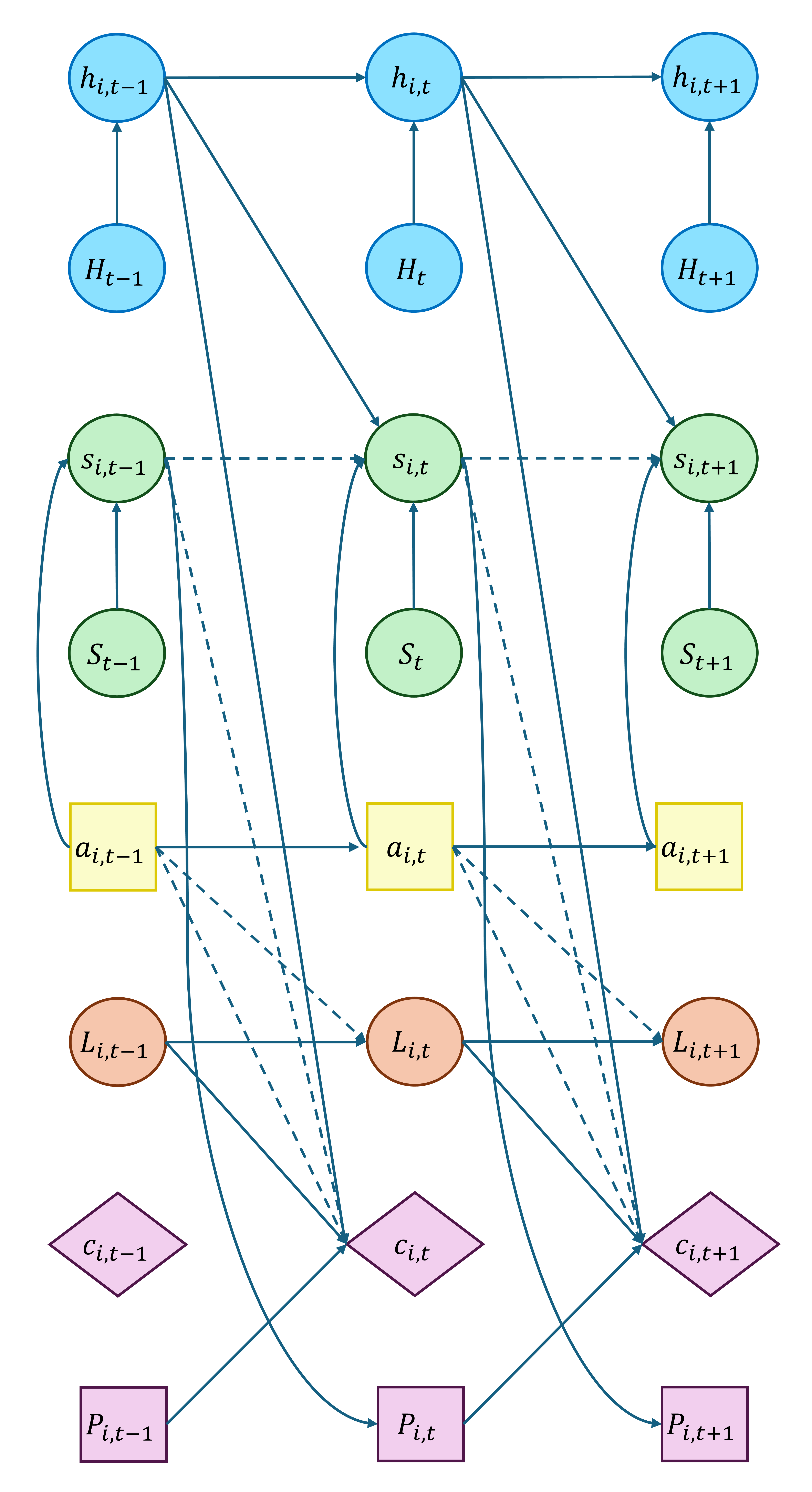
***

*Figure S4. Gray whale model represented as a directed acyclic graph. For simplicity, only three time steps (t – 1, t, and t + 1) in the time series for individual i are reported. The shapes and colours used to represent the variables mimic the model diagram provided in the main text (Fig. 1), but note that, for clarity, we do not report the observation models that generate the data. Hypothesised causal relationships between variables are represented as solid lines, while dashed lines indicate causal relationships for which we found no support in preliminary analyses (e.g., the effect of prior stress state on current stress state) or that are described in previous studies from which we derived the input data (e.g., the relationship between age and body length). Model variables include: nutritional state (h_i,t_), population mean nutritional state (H_t_), stress state (s_i,t_), population mean stress state (S_t_), age (a_i,t_), length (L_i,t_), calving status (c_i,t_), and pregnancy status (P_i,t_) (for simplicity, we do not report sex and its effect on the stress state). Note that we represent the effect of stress state on calving via the hypothesised causal pathway (i.e., through the effect of stress on pregnancy in the next year, which in turn determines whether the female gives birth to a calf two years later); in practice, given the available data, we modelled the effect of stress state on calving probability with a two-year lag. Moreover, here we represent the mechanistic relationship between pregnancy status and calving status (i.e., a female is pregnant and then gives birth to a calf), even though in the model we inform pregnancy status from calving status in the following year (as represented in the model diagram in Fig. 1).*

Prior distributions and posterior estimates of all model parameters

*Table S1. Prior distribution and posterior mean, standard deviation (SD), and 2.5^th^, 50^th^ (median) and 97.5^th^ quantiles of model parameters. Note that we use non-informative priors for all parameters, except for the standard deviation of the nutritional state process (which was constrained as discussed in the note below) and for the effects of covariates on calving probability (which were centred on a positive or negative value according to prior expectations; see main text and section ‘Investigating the sensitivity of the results to the priors for the calving probability model’ for discussion).*

| **Parameter** | **Symbol** | **Prior** | **Posterior** | | | | |
| --- | --- | --- | --- | --- | --- | --- | --- |
|  |  |  | *Mean* | *SD* | *2.50%* | *50%* | *97.50%* |
| Effect of nutritional state in the previous time step | $\beta_{h}$ | *Normal*(0, 1) | 0.39 | 0.12 | 0.14 | 0.40 | 0.61 |
| Mean of population-level random effect of year on nutritional state | *λ_h_* | *Normal*(28, 5) | 27.85 | 0.53 | 26.79 | 27.84 | 28.96 |
| Standard deviation of population-level random effect of year on nutritional state | *χ_h_* | *Uniform*(0, 5) | 1.36 | 0.54 | 0.67 | 1.23 | 2.73 |
| Standard deviation of nutritional state process | *σ_h_* | *Uniform*(*ν_h_*, 5)^*^ | 1.65 | 0.06 | 1.54 | 1.65 | 1.78 |
| Effect of the number of days between measurement date and the end of the field season on BAI | $\delta_{1}$ | *Normal*(0, 1) | -0.02 | 0.00 | -0.02 | -0.02 | -0.01 |
| Effect of pregnancy state on BAI | $\delta_{2}$ | *Normal*(0, 1) | 0.68 | 0.58 | -0.35 | 0.63 | 1.95 |
| Standard deviation of BAI observation model | *ν_h_* | *Uniform*(0, 5) | 1.61 | 0.05 | 1.51 | 1.61 | 1.71 |
| Effect of nutritional state at the end of the previous feeding season on stress state | $\beta_{s, 1}$ | *Normal*(0, 1) | -0.09 | 0.07 | -0.22 | -0.09 | 0.06 |
| Effect of age on stress state | $\beta_{s, 2}$ | *Normal*(0, 1) | -0.02 | 0.05 | -0.12 | -0.02 | 0.08 |
| Effect of sex on stress state | $\beta_{s, 3}$ | *Normal*(0, 1) | 0.26 | 0.12 | 0.03 | 0.26 | 0.49 |
| Mean of population-level random effect of year on stress state | *λ_s_* | *Normal*(2, 1) | 0.49 | 0.39 | -0.27 | 0.49 | 1.25 |
| Standard deviation of population-level random effect of year on stress state | *χ_s_* | *Uniform*(0, 5) | 0.77 | 0.29 | 0.42 | 0.71 | 1.54 |
| Standard deviation of stress state process | *σ_s_* | *Uniform*(0, 5) | 0.40 | 0.06 | 0.29 | 0.40 | 0.51 |
| Effect of concentration of progestin metabolites on faecal glucocorticoid concentration | $\zeta_{1}$ | *Normal*(0, 1) | 0.30 | 0.05 | 0.21 | 0.30 | 0.40 |
| Effect of concentration of androgen metabolites on faecal glucocorticoid concentration | $\zeta_{2}$ | *Normal*(0, 1) | 0.18 | 0.04 | 0.09 | 0.18 | 0.26 |
| Effect of concentration of thyroid hormone metabolites on faecal glucocorticoid concentration | $\zeta_{3}$ | *Normal*(0, 1) | 0.23 | 0.03 | 0.18 | 0.23 | 0.30 |
| Effect of day of the year on faecal glucocorticoid concentration | $\zeta_{4}$ | *Normal*(0, 1) | -0.003 | 0.001 | -0.005 | -0.003 | -0.001 |
| Standard deviation of faecal glucocorticoid concentration observation model | *ν_s_* | *Uniform*(0, 10) | 6.45 | 0.32 | 5.85 | 6.44 | 7.12 |
| Intercept of calving probability model | $\gamma_{1}$ | *Normal*(0, 1.5) | -2.25 | 0.90 | -3.90 | -2.28 | -0.40 |
| Effect of the standardised nutritional state in the previous time step on calving probability | $\gamma_{2}$ | *Normal*(1, 1) | 0.90 | 0.64 | -0.11 | 0.80 | 2.38 |
| Effect of the standardised cube of length in the previous time step on calving probability | $\gamma_{3}$ | *Normal*(1, 1) | 1.38 | 0.61 | 0.29 | 1.36 | 2.64 |
| Effect of the residual of stress state two years prior on calving probability | $\gamma_{4}$ | *Normal*(-1, 1) | -1.00 | 0.73 | -2.48 | -0.97 | 0.39 |
| Intercept of the observation model for calving probability | $\eta_{1}$ | *Normal*(1, 1) | 0.55 | 0.75 | -0.75 | 0.48 | 2.13 |
| Effect of day of first sighting of a female on the probability of observing a calf | $\eta_{2}$ | *Normal*(-1, 1.5) | -1.82 | 1.03 | -3.86 | -1.80 | 0.16 |

^*^ Note that we constrain the standard deviation of the nutritional state process (i.e., the variation around the expected nutritional state in a given feeding season) to be larger than the observation error around BAI. This is because the deviation from the expected value (which emerges from the population-level mean and the effect of the individual’s state in the previous year) is likely to be driven by other covariates that are currently not included in the process model, rather than by uncertainty in the observations.

Model fitting and diagnostics

The model was fitted in a Bayesian framework using Markov Chain Monte Carlo algorithms in package NIMBLE (de Valpine et al., 2017) for R (R Core Team, 2023). Three chains were run for 350,000 iterations, after discarding 50,000 iterations as burn-in. One in 350 iterations were retained for posterior inference, to reduce object sizes. Chain convergence and mixing were assessed visually using trace plots, as well as via the Brooks-Gelman-Rubin diagnostic (< 1.1), Monte Carlo error (< 5% of the sample standard deviation), and effective sample size (≥ 400) (Lunn et al., 2013). We assessed the goodness-of-fit of the calving model by comparing the combined probability of observing calves in a given year ($c_{i,t}p_{i,t} e_{i,t}$) with calf observations in our study area (*k_i,t_*). Specifically, a receiver operating characteristic (ROC) curve was used to select a cut-off for classifying combined probabilities as either 1s or 0s, and a confusion matrix was used to compare binary predictions with observed values. The area under the ROC curve (AUC) was used as a metric of overall fit.

Extension of the growth model by Pirotta et al. (2024) to estimate body area index (BAI)

Pirotta et al. (2024) proposed an approach to model the growth of individual whales, while accounting for the uncertainty associated with photogrammetric estimates derived from drone imagery. The approach was developed using a subset of the morphometric data that is presented in this work (2016-2022). Their model can be readily extended to estimate other metrics derived from aerial images, such as body area index (BAI), and propagate the various uncertainty components appropriately.

BAI is defined as:

$$\mathrm{BAI}=\frac{SA}{{(r\cdot L)}^{2}} \cdot100$$

Where *SA* is surface area, calculated as $SA= \sum_{s=1}^{S} \frac{0.05 L}{2}(W_{s}+W_{s+1})$, *r* is the proportion of the body covered by the surface area (e.g., 0.5 if calculating BAI between 20% and 70% body length), and *L* is total length (Bierlich et al., 2021; Burnett et al., 2019). *SA* is the sum of the areas between consecutive body widths (*W*), measured at 5% increments of *L*. The estimation of BAI therefore requires the estimation of width at different positions along the body, from the corresponding pixel measurements.

Building on the approach by Pirotta et al. (2024), we model each width pixel measurement at a given 5% increment *s* as: $X_{s}^{w}\sim Normal(P_{s}^{w}, \sigma_{P})$, where $\sigma_{P}$ is pixel measurement error, and $P_{s}^{w}$ is the true width in pixels. The latter is modelled as a proportion of the pixel measurement of total length ($P^{l}$), i.e., $P_{s}^{w}=P^{l} p_{s}$, where $p_{s}$ is a proportion with prior *Uniform*(0.001, 0.5). Consequently, the true width in meters at each increment can be calculated as $W_{s}=L p_{s}$, and then used in the calculation of BAI.

Temporal lag between the residual of the stress state and calving probability

Chronic stress is known to have a suppressive effect on reproduction (e.g., Rivier & Rivest, 1991). This effect could manifest at different stages of the reproductive process, e.g., it could prevent the initiation of pregnancy or it could interfere with the healthy development of the foetus during gestation. For example, among marine mammals, cortisol was documented to modulate the molecular pathway leading to implantation in northern elephant seals (*Mirounga angustirostris*) (Sperou, 2020). Therefore, multiple time lags are possible between the residual of the stress state in our model (which we take to represent an individual’s average chronic stress level in a feeding season) and the probability of a female to produce a calf in a given year.

Here, we investigated a one-year time lag between the stress state residual and a female’s calving probability, in addition to the two-year lag we present in the main text. The median estimated effect of the residual stress state in the previous time step was smaller, and its 95% credible intervals showed a larger overlap with 0 (*γ*_4_ = -0.53 [-2.06 – 0.71]; Fig. S5). These results suggest that a two-year time lag provides a better representation of the relationship between the residual stress state and calving probability, reinforcing the potential role of stress on the initiation of pregnancy.


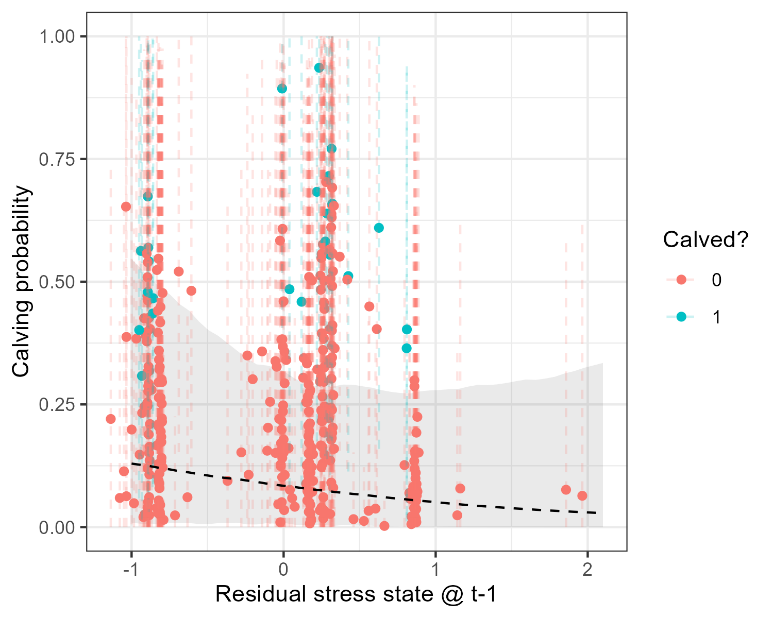


*Figure S5. Residual stress state in the year prior to a reproductive opportunity, plotted against the estimated calving probability (the dots indicate the posterior medians, while the dashed segments are the 95% credible intervals), and coloured by whether the model estimated an individual to have calved (blue) or not (red). The black dashed lines and grey ribbons represent the estimated relationship (median and 95% credible intervals) between this covariate and calving probability, with standardised nutritional state and cubed body length set to their mean.*

Alternative model formulation excluding the stress state process

The results presented in the main text suggest that the stress state process is associated with the greatest level of uncertainty in the model, due in part to limitations imposed by sample size. In particular, we show how the relatively small number of repeated faecal samples for individual whales resulted in a smaller deviation of individual stress state from the population’s mean in a given year, as compared to individual variation around the nutritional state process. Here, we investigate the results of an alternative model formulation where the stress state process is not modelled, i.e., a model that only focused on nutritional state, body length, and their effect on calving probability.

The estimated median effects of nutritional state and the cube of length in the previous time step on calving probability were comparable to the effects estimated in the model presented in the main text, albeit with a slightly narrower 95% credible intervals (*γ*_2_ = 0.76 [-0.07 – 2.25], *γ*_3_ = 1.28 [0.32 – 2.53]). However, we note that the number of additional calving events that were estimated to have been missed over the study period is much smaller (*n* = 3, as compared to *n* = 19 in the model presented in the main text), which is the result of a smaller estimated calving probability on average (Fig. S6).


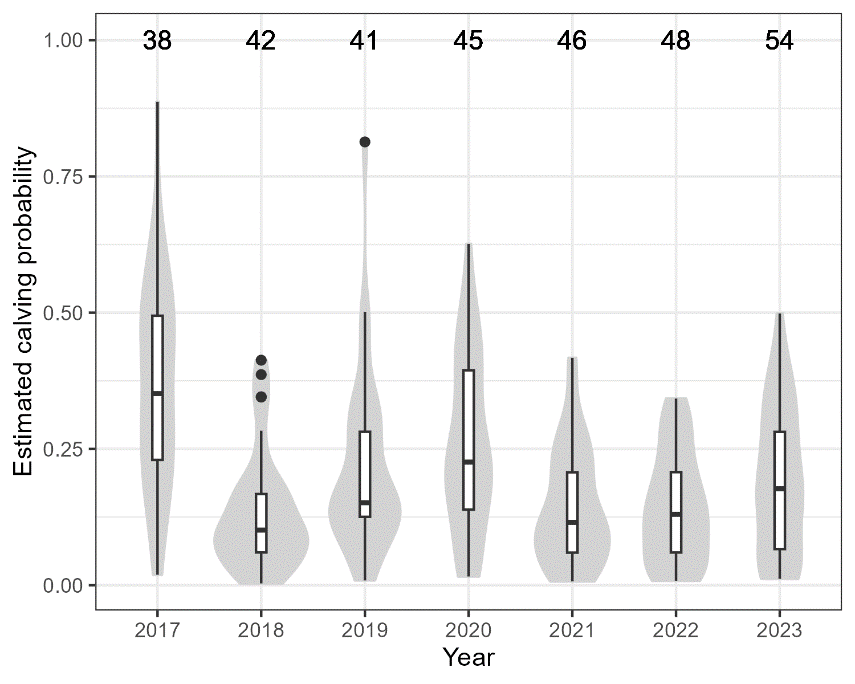


*Figure S6. Distribution of estimated calving probabilities across females in the model without the stress state process (i.e., only including the effects of nutritional state and body length on calving probability). At the top of the plot, the number of females available to reproduce in each year (i.e., 8 y or older, and non-pregnant) is reported.*

Investigating the sensitivity of the results to the priors for the calving probability model

As discussed in the main text, several mechanistic assumptions were required in order to successfully fit the model to the available data. For example, the prior distributions for the effects of nutritional state, the cube of length and the residual of stress state on calving probability (i.e., the *γ*_2_, *γ*_3_, and *γ*_4_ parameters) were normal distributions centred on a mean value that reflected our expectation for the direction of those relationships (Table S1). Even though the standard deviations of those priors were large (see Table S1), model results could still be sensitive to this model specification.

Here, we investigate this prior sensitivity by refitting the model using priors for the *γ* parameters centred on 0, i.e., *Normal*(0, 1). The results suggest that the posterior estimates are partly sensitive to the specification of these priors: while the posterior means were still centred on values that were mechanistically reasonable and in line with our interpretation in the main text (that is, a positive effect of the cube of length and nutritional state in the previous year, and a negative effect of the residual stress state two years prior), the median effects were smaller and the 95% credible intervals showed a greater overlap with 0 (*γ*_2_ = 0.51 [-0.28 – 1.70], *γ*_3_ = 0.96 [-0.01 – 2.09], *γ*_4_ = -0.44 [-1.76 – 0.78]). As a result, the number of calving events that were estimated to have been missed was also smaller (*n* = 4). These results reinforce the conclusion that, due to the short time series of data, the mechanistic assumptions that underpin the modelling have a considerable influence on model results. As the dataset expands, these assumptions should be regularly re-evaluated to ensure that they are supported by the growing number of observations.

Using a restricted estimate of body area index (BAI)

Pre-pregnant female baleen whales have been shown to accumulate lipid reserves in preparation for supporting the energetic costs of gestation and lactation (Miller et al., 2011). However, during pregnancy, the shape of a female’s body may also change due to the growing foetus, with consequences for the interpretation of drone-derived measures of body condition (Miller et al., 2012). In the model presented in the main text, we included the effect of pregnancy status on the measurement of nutritional state via BAI. This effect was estimated to be centred on a positive value, but it is possible that the effect of nutritional state (measured through BAI) on calving probability was still partially confounded with the effect of pregnancy on body shape.

Here, we investigate an alternative way to compute relative body condition, which would allow us to remove the effect of pregnancy on the observation process. Specifically, we restricted the calculation of BAI to the frontal section of the body, where changes in body width are unlikely to be affected by the presence of the foetus (i.e., along the rib cage). The calculation of surface area was limited between 20% and 40% of a whale’s total length (as opposed to the head-tail range, covering 20-70% of a whale’s length, used in the standard calculation of BAI; Fig. S7) (Torres et al., 2022), and the effect of pregnancy was then dropped from the observation model. The results of this model indicated that nutritional state in the previous time step was still associated with the probability of calving; in fact, the posterior median effect was greater than in the model presented in the main text, and the posterior 95% credible intervals did not overlap with 0 (*γ*_2_ = 1.52 [0.25 – 2.93]).

While body width in the frontal part of the body is less variable following changes in body condition (Bierlich et al., 2021; Fernandez Ajó et al., 2023), restricting the calculation of body condition metrics to this area could obviate the need to correct for the effect of pregnancy on body shape. Future work should further investigate this option, e.g., via an in-depth exploration of the temporal variation in frontal BAI, and of the correlation among alternative BAI calculations for animals in different reproductive states.

***
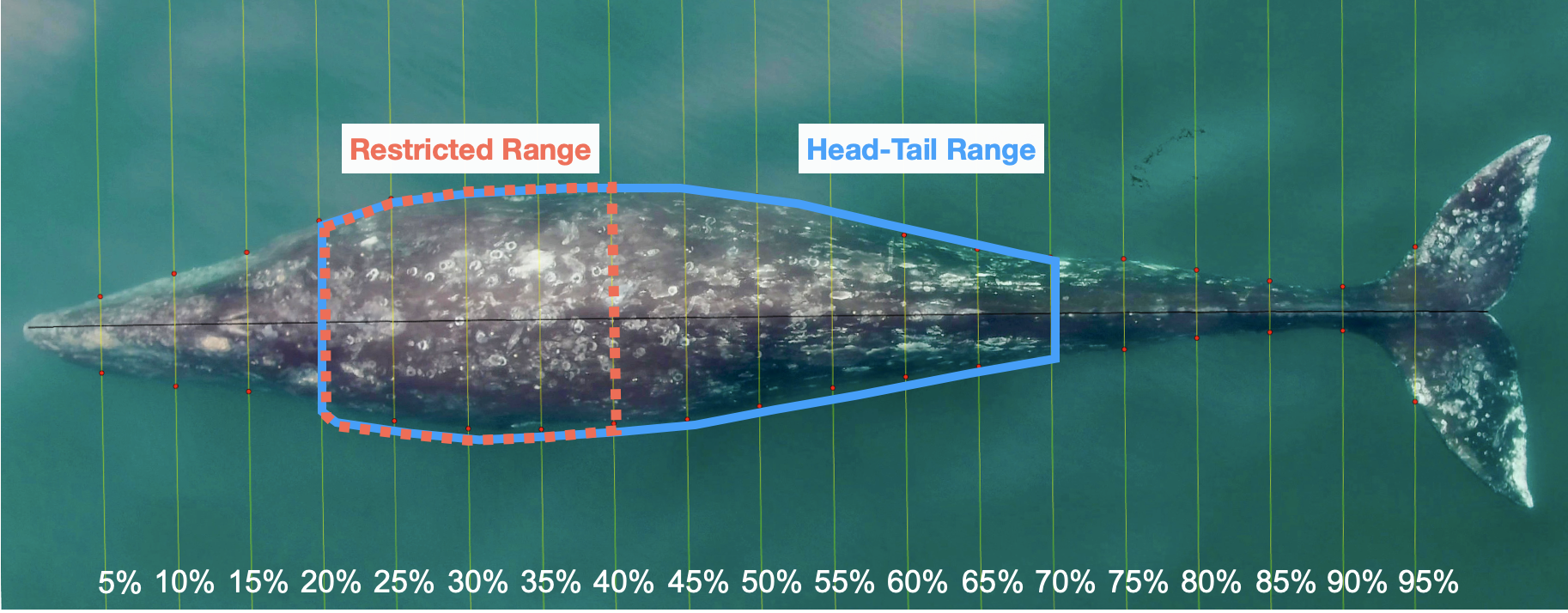
***

*Figure S7. A drone image of a gray whale, highlighting the region of the body used to calculate the standard BAI (i.e., the head-tail range, including perpendicular widths in 5% increments of total length between 20% and 70%) and the restricted 20-40% range that was investigated here.*

Investigating the effect of age on calving probability

Body length shows high collinearity with age, because animals increase in body length as they age. As a result of this dependency, the relationship we estimated between length and calving probability could act as a proxy of a female having to reach a certain age before she can attempt to reproduce (but note that sexual maturity could itself be determined by having achieved a sufficient body size to support a reproductive attempt, i.e., it could be dependent on growth). However, our model formulation does not allow the investigation of any residual effect of age beyond the time when a female has reached her asymptotic size, e.g., the occurrence of senescence.

Here, we explore the evidence for these residual effects. To avoid any issue associated with the collinearity between length and age, we allowed the effect of age to start contributing to calving probability after female length has stabilised, i.e., after females have approximately reached their asymptotic size. Given the estimated growth curve in Pirotta et al. (2024), growth in length appears to be limited after the age of 20 y across individuals. Therefore, we formulated a new version of the model where age can potentially affect calving probability after age 20 y (note that data availability did not support the direct estimation of this threshold). Specifically, we included an additional covariate (with coefficient *γ*_5_), which was multiplied by a binary variable that took value of 0 before age 20 y, and value of 1 from age 20 y onwards.

The posterior distribution of *γ*_5_ overlapped with 0 (median: 0.49; 95% credible interval: -0.74 – 1.69), although there was a 0.8 probability that the effect was positive. Therefore, we did not find any evidence of senescence based on the available data. In contrast, this result could suggest that calving probability continues increasing after the age of maturity, potentially associated with a female’s increasing experience. However, it should be noted that older females also tend to be asymptotically longer (Pirotta et al., 2024), so this result could emerge from a confounded effect with length; more data are needed to tease these two possible explanations apart. The inclusion of the effect of age as described above did not alter the estimates of the other parameters in the model for calving probability, suggesting that the threshold formulation was successfully avoiding any issues with collinearity.

References

Bierlich, K. C., Hewitt, J., Bird, C. N., Schick, R. S., Friedlaender, A., Torres, L. G., Dale, J., Goldbogen, J., Read, A. J., Calambokidis, J., & Johnston, D. W. (2021). Comparing uncertainty associated with 1-, 2-, and 3D aerial photogrammetry-based body condition measurements of baleen whales. *Frontiers in Marine Science*, *8*, 1–16. https://doi.org/10.3389/fmars.2021.749943

Burnett, J. D., Lemos, L., Barlow, D., Wing, M. G., Chandler, T., & Torres, L. G. (2019). Estimating morphometric attributes of baleen whales with photogrammetry from small UASs: A case study with blue and gray whales. *Marine Mammal Science*, *35*(1), 108–139. https://doi.org/10.1111/mms.12527

de Valpine, P., Turek, D., Paciorek, C. J., Anderson-Bergman, C., Temple Lang, D., & Bodik, R. (2017). Programming with models: writing statistical algorithms for general model structures with NIMBLE. *Journal of Computational and Graphical Statistics*, *26*, 403–413. https://doi.org/10.1080/10618600.2016.1172487

Fernandez Ajó, A., Pirotta, E., Bierlich, K. C., Hildebrand, L., Bird, C. N., Hunt, K. E., Buck, C. L., New, L., Dillon, D., & Torres, L. G. (2023). Assessment of a non-invasive approach to pregnancy diagnosis in gray whales through drone-based photogrammetry and faecal hormone analysis. *Royal Society Open Science*, *10*(7), 230452. https://doi.org/10.1098/rsos.230452

Lunn, D., Jackson, C., Best, N., Thomas, A., & Spiegelhalter, D. (2013). *The BUGS book: A practical introduction to Bayesian analysis*. Chapman & Hall/CRC.

Miller, C., Best, P., Perryman, W., Baumgartner, M., & Moore, M. (2012). Body shape changes associated with reproductive status, nutritive condition and growth in right whales *Eubalaena glacialis* and *E. australis*. *Marine Ecology Progress Series*, *459*, 135–156. https://doi.org/10.3354/meps09675

Miller, C., Reeb, D., Best, P., Knowlton, A., Brown, M., & Moore, M. J. (2011). Blubber thickness in right whales *Eubalaena glacialis* and *Eubalaena australis* related with reproduction, life history status and prey abundance. *Marine Ecology Progress Series*, *438*, 267–283. https://doi.org/10.3354/meps09174

Pirotta, E., Bierlich, K. C., New, L., Hildebrand, L., Bird, C. N., Fernandez Ajó, A., & Torres, L. G. (2024). Modeling individual growth reveals decreasing gray whale body length and correlations with ocean climate indices at multiple scales. *Global Change Biology*, *30*, e17366. https://doi.org/10.1111/gcb.17366

R Core Team. (2023). *R: A language and environment for statistical computing. R Foundation for Statistical Computing, Vienna, Austria. URL http://www.R-project.org/.*

Rivier, C., & Rivest, S. (1991). Effect of stress on the activity of the hypothalamic-pituitary-gonadal axis: peripheral and central mechanisms. *Biology of Reproduction*, *45*, 523–532.

Sperou, E. S. (2020). *A comprehensive analysis of sex hormones and their role in reproductive suppression in female northern elephant seals (Mirounga angustirostris). MSc thesis, Sonoma State University.*

Torres, L. G., Bird, C. N., Rodríguez-González, F., Christiansen, F., Bejder, L., Lemos, L., Urban R, J., Swartz, S., Willoughby, A., Hewitt, J., & Bierlich, K. C. (2022). Range-wide comparison of gray whale body condition reveals contrasting sub-population health characteristics and vulnerability to environmental change. *Frontiers in Marine Science*, *9*, 511. https://doi.org/10.3389/fmars.2022.867258
